# The Association of Body Mass Index and Body Composition with Pain, Disease Activity, Fatigue, Sleep and Anxiety in Women with Fibromyalgia

**DOI:** 10.3390/nu11051193

**Published:** 2019-05-27

**Authors:** María Correa-Rodríguez, Jamal El Mansouri-Yachou, Antonio Casas-Barragán, Francisco Molina, Blanca Rueda-Medina, María Encarnación Aguilar-Ferrándiz

**Affiliations:** 1 Department of Nursing, Faculty of Health Sciences, University of Granada (UGR), Instituto de Investigación Biosanitaria Granada (IBS Granada), 18016 Granada, Spain; macoro@ugr.es (M.C.-R.); blarume@ugr.es (B.R.-M.); 2 Department of Physical Therapy, Faculty of Health Science, University of Granada (UGR), 18016 Granada, Spain; jmans@outlook.es (J.E.M.-Y.); antoniocb@ugr.es (A.C.-B.); 3 Department of Health Science, University of Jaén, Paraje Las Lagunillas s/n, 23071 Jaén, Spain; 4 Department of Physical Therapy, Faculty of Health Science, University of Granada (UGR), Instituto de Investigación Biosanitaria Granada (IBS. Granada), 18016 Granada, Spain; e_aguilar@ugr.es

**Keywords:** body mass index, fat mass, tender point counts, visual analog scale, disease severity, sleep, anxiety, fibromyalgia

## Abstract

The link between fibromyalgia syndrome (FMS) and obesity has not been thoroughly investigated. The purpose of this study was to examine the relationships among body mass index (BMI) and body composition parameters, including fat mass, fat mass percentage, and visceral fat, as well as FMS features, such as tender point count (TPC), pain, disease activity, fatigue, sleep quality, and anxiety, in a population of FMS women and healthy controls. A total of seventy-three women with FMS and seventy-three healthy controls, matched on weight, were included in this cross-sectional study. We used a body composition analyzer to measure fat mass, fat mass percentage, and visceral fat. Tender point count (TPC) was measured by algometry pressure. The disease severity was measured with the Fibromyalgia Impact Questionnaire (FIQ-R) and self-reported global pain was evaluated with the visual analog scale (VAS). To measure the quality of sleep, fatigue, and anxiety we used the Pittsburgh Sleep Quality Questionnaire (PSQI), the Spanish version of the multidimensional fatigue inventory (MFI), and the Beck Anxiety Inventory (BAI), respectively. Of the women in this study, 38.4% and 31.5% were overweight and obese, respectively. Significant differences in FIQ-R.1 (16.82 ± 6.86 vs. 20.66 ± 4.71, *p* = 0.030), FIQ-R.3 (35.20 ± 89.02 vs. 40.33 ± 5.60, *p* = 0.033), and FIQ-R total score (63.87 ± 19.12 vs. 75.94 ± 12.25, *p* = 0.017) among normal-weight and overweight FMS were observed. Linear analysis regression revealed significant associations between FIQ-R.2 (β(95% CI) = 0.336, (0.027, 0.645), *p* = 0.034), FIQ-R.3 (β(95% CI) = 0.235, (0.017, 0.453), *p* = 0.035), and FIQ-R total score (β(95% CI) = 0.110, (0.010, 0.209), *p* = 0.032) and BMI in FMS women after adjusting for age and menopause status. Associations between sleep latency and fat mass percentage in FMS women (β(95% CI) = 1.910, (0.078, 3.742), *p* = 0.041) and sleep quality and visceral fat in healthy women (β(95% CI) = 2.614, (2.192, 3.036), *p* = 0.008) adjusted for covariates were also reported. The higher BMI values are associated with poor FIQ-R scores and overweight and obese women with FMS have higher symptom severity. The promotion of an optimal BMI might contribute to ameliorate some of the FMS symptoms.

## 1. Introduction

Fibromyalgia syndrome (FMS) is a functional syndrome characterized by widespread pain and several associated symptoms including cognitive dysfunction, fatigue, sleep disorders, reduced pain threshold, and morning stiffness [1]. The etiopathogenetic of widespread pain in FMS is yet to be elucidated [2]. Abnormal endogenous pain modulation [3], reduced hypothalamic-pituitary-adrenal axis (HPA) activity [4], and immune system abnormalities [5] have been proposed as contributing factors to FMS.

The link between FMS and obesity has not been thoroughly investigated [6]. However, the aforementioned dysfunctions have also been observed in obesity, a complex disorder defined as excessive fat accumulation in adipose tissue [7]. It has been reported that obese individuals experimented a decreased pain threshold to electrical or mechanical stimuli [8,9]. A large-scale survey study with over 1 million people in the US showed a linear increment of chronic pain cases as body mass index (BMI) increases [10]. In addition, previous findings indicated that obesity is associated with altered HPA activity [11]. Immune cells also play a main role in inducing low-grade chronic inflammation in obesity [12]. In fact, obesity has been associated with elevated levels of proinflammatory markers such as interleukins, C reactive protein (CRP), interferon (IFN)-γ, and tumor necrosis factor (TNF)-α [13,14].

Previous studies have shown that the prevalence of overweight and obesity is high in FMS patients, ranging from 62% to 73% [8,15,16,17,18,19]. Although increased BMI has been associated with multiple pain measures, symptom severity, disease activity, fatigue, anxiety, or quality of life in FMS patients, the results are still controversial [8,15,16,17,18,19,20,21,22,23,24]. Whereas, in some studies, obesity has been related to numerous FMS-related symptoms, other authors have reported a lack of association [8,16,17,18,20,25,26]. These contradictory results observed may be explained by the differences in sample characteristics, such as age range or ethnicity, and methodological differences in the assessment of FMS symptoms. Therefore, further studies are needed to further evaluate the association between obesity and FMS.

Nonrestorative sleep is a common problem in FMS patients [27,28]. Interestingly, it has been reported that women with sleep problems had significantly higher pain scores on the tender point index, and they reported significantly more symptoms of depressive and a more negative impact of FMS on functioning than those without sleep deficits [29]. Similarly, women with FMS showed poorer sleep quality and more fatigue as compared with controls, supporting the finding that self-reported sleep quality and fatigue are associated with behavioral indicators of sleep quality in FMS women [30]. Considering the relevance of sleep problems in FMS, a recent study has proposed a machine learning method for detecting extreme cases of poor sleep and fatigue in FMS patients [31].

On the basis of the data evidence, it is suggested that optimal body weight may be one of the main factors in the management of FMS symptomatology [8,16,17,18,20,25,26]. However, previous work has included only BMI as a marker of obesity. The role of body composition measurements including fat mass, fat mass percentage, and visceral fat have not been widely examined and, to our knowledge, only one study has investigated the role of body fat mass on FMS features [25]. Body composition measurements might provide data to further understand the associations between obesity and FMS.

Given that (1) a main goal in the management of FMS patients is the improvement of quality of life by ameliorating clinical symptomatology and (2) previous work has shown a high prevalence of overweight and obesity among FMS women, and therefore investigating the associations among obesity measurements and numerous FMS-related symptoms is of special interest. The purpose of this study was to examine the relationship between BMI and body composition parameters, including fat mass, fat mass percentage, and visceral fat, and FMS features, such as tender point count (TPC), pain, disease activity, fatigue, sleep quality, and anxiety, in a population of FMS women and healthy controls.

## 2. Materials and Methods

### 2.1. Study Population

A total of seventy-three women diagnosed with FMS according to the criteria of the American College of Rheumatology (ACR) of 1990 and seventy-three healthy controls matched on weight, aged 30 and 70 years, were enrolled in this case-control study. We decided to perform this study only on women because FMS is most prevalent among middle-aged women, encompassing 75–90% of those diagnosed [32,33]. The higher frequency of FMS among women has been attributed to the fact that women tend to report more tender points than men [34] and feel pain more intensely at these sites [35]. Women with FMS were identified from the Granada Fibromyalgia Association (AGRAFIM, Spain) and Jaén Fibromyalgia Association (AFIXA). We recruited controls from friends and relatives of the patients, friends, and colleagues of controls, and the Faculty of Health Sciences (University of Granada) employees. The participants completed structured questionnaires regarding their medical history, medications, age of fibromyalgia diagnosis, and menopause status. The exclusion criteria included any medical condition that affected body weight such as a history of psychiatric illness, autoimmune disease, diabetes mellitus, thyroid dysfunction, as well as active infections, pregnancy, and breastfeeding. Women who were on antidepressant medication or taking sleeping pills also were excluded. Only one study visit was required for each subject. During the visit, informed consent was obtained, the objective of the study was explained, and any questions were answered. The study was approved by the Ethics Committee of the University of Granada. This research was performed in strict compliance with the international code of medical ethics established by the World Medical Association and the Declaration of Helsinki.

### 2.2. Body Composition Measurements

A body composition analyzer (TANITA BC-418MA^®^) was used to measure the percentage of fat mass, fat mass (kg), and visceral fat (without shoes and in light clothes) to the nearest 0.1 kg. Height was measured using a Harpenden stadiometer (Holtain 602VR^®^) to the nearest 0.5 cm, with participants again not wearing shoes. BMI was calculated by dividing weight and height squared (kg/m^2^). Body weight and height were measured twice. The average of each measure was used for the analysis. The same trained research assistant performed all the measurements.

### 2.3. Pressure Pain Threshold (PPT) and Tender Point Counts (TPC)

Algometry is a quantitative method for the assessment of tenderness commonly used in clinical practice [36]. PPT is defined as the minimal amount of pressure where a sensation of pressure first changes to pain [37]. A digital pressure algometer was used in this study. The device consisted of a 1 cm^2^ rubber disk attached to a strain gauge which displayed values in kPa (Storz Medical AG, Tagerwilen, Switzerland). Participants’ PPTs were determined by gradually increasing the pressure applied by the algometer (at a rate of 1 kg/s) until the point when the sensation first became painful (participants were instructed to say “stop” at this point). The mean of 3 trials was calculated and used for the main analysis. A 30-s resting period was allowed between each recording. The reliability of pressure algometry was found to be high the same day (intraclass correlation coefficient, 0.91) [36] and between 4 separate days (intraclass correlation coefficient, 0.94–0.97) [38]. The PPT was assessed bilaterally over the 18 tender points considered by the American College of Rheumatology for FMS diagnosis. A tender point was considered positive if participants reported “pain” at or below a pressure of 4 kg. The total of such positive tender points was recorded as the individual’s TPC. The maximum score for TPC was 18.

### 2.4. Visual Analog Scale (VAS)

The global pain of the patient was assessed using the visual analog scale (VAS) pain score 0–10 cm, with higher scores indicating more pain. The VAS was shown to be an important instrument in pain evaluation, being sensitive and specific in the assessment of pain in FMS [39].

### 2.5. Fibromyalgia Impact Questionnaire (FIQ-R)

The Spanish version of the FIQ-R was used to assess the impact of FMS symptoms on the physical and mental health of patients. This self-reported questionnaire consisted of twenty-one domains assessing physical impairment, number of days feeling good, work missed, ability to do work, pain, fatigue, rested, stiffness, anxiety, and depressive symptoms. The total score came from the sum of all subscales, where higher scores indicated a negative impact (0 to 100) [40].

### 2.6. Sleep Quality Index

The validated version of the Pittsburgh Sleep Quality Questionnaire (PSQI) for the Spanish population was performed to evaluate the quality of sleep [41]. It consisted of 24 items, where the patients responded to 19 of these items and a person who lived in the same home (or hospital room) responded to the remaining 5. In this questionnaire the following 7 subdimensions were evaluated: subjective quality of sleep, sleep latency, sleep duration, sleep efficiency, sleep disturbances, sleep medication, and diurnal dysfunction. Each dimension was scored from 0 points (no problem) to 3 points (serious problem), where the total score varied in a range from 0 to 21 points. Higher scores reported a poorer quality of sleep. The PSQI showed high reliability with a Cronbach’s alpha of 0.805 [41].

### 2.7. General Self-Perceived Fatigue

The Spanish version of the multidimensional fatigue inventory (MFI) was used to evaluate fatigue severity of patients with FMS [42]. This questionnaire contained the following 5 subscales: general fatigue, physical fatigue, mental fatigue, reduced activity, and reduced motivation. Each subscale included 4 questions with a score from 1 point to 5 points, where high scores indicated a higher degree of fatigue. The test–retest analysis of reliability showed an excellent domains correlation that ranged from 0.64 to 0.91 [42].

### 2.8. Anxiety

The Beck Anxiety Inventory (BAI) was used to evaluate the psychological aspects and common symptoms of anxiety [43]. This questionnaire contained 21 items that assessed the severity of patient anxiety with a score range from 0 points (nothing anxiety) up to 3 points (a lot of anxiety). The total scores ranged from 0 to 63 points, where high scores indicated a higher degree of anxiety (17). The test–retest reliability analysis of the Spanish version showed high internal consistency with a Cronbach alpha of 0.91 [44].

### 2.9. Statistical Analysis

Data were analyzed using SPSS^©^ version 22.0 (IBM Corporation, Armonk, NY, USA). The Kolmogorov–Smirnov test was used to analyze the normality of the distribution of the variables (*p* > 0.05) [45]. To compare the two groups, we used the Mann–Whitney U test and Student’s *t*-test for continuous data and X^2^ for categorical data. The differences in clinical variables between normal-weight and overweight/obese women with FMS and healthy women were determined using analysis of covariance (ANCOVA) after adjusting for age and menopausal state [46]. Linear regression analyses were conducted to determine the associations among body composition status and VAS, TPC, FIQ-R, fatigue, sleep, and anxiety, after adjusting by age and menopausal state. The results were reported as a percentage change (β) with 95% confidence intervals (95% CI) and *p*-values of < 0.05 were considered to be statistically significant.

## 3. Results

### 3.1. Demographic and Clinical Characteristics

The demographic and clinical characteristics of women with FMS and healthy controls are shown in Table 1. The mean age of the study population was 56.96 ± 9.23 years. On the basis of the BMI classification, 39.0% and 29.5% of the study women were overweight and obese, respectively. Note that the mean BMI was within the overweight range (28.66 ± 5.36 kg/m^2^). No significant differences were observed between FMS patients and healthy women with respect to height, weight, BMI, fat mass, fat mass percentage, and visceral fat. However, the mean and standard deviation of TPC, VAS, fatigue, sleep quality, and anxiety were significantly higher in FMS patients than in the controls (*p* < 0.05). The mean of the total score of FIQ-R was 73.08 ± 14.73.

### 3.2. Comparison of Clinical Variables between Normal-Weight and Overweight/Obese Women with FMS and Healthy Controls

Table 2 shows the comparison of clinical variables between the normal-weight and overweight/obese FMS women and the healthy controls. For the FMS women, significant differences in FIQ-R.1(16.82 ± 6.86 vs. 20.66 ± 4.71, *p* = 0.030), FIQ-R.3 (35.20 ± 89.02 vs. 40.33 ± 5.60, *p* = 0.033) and FIQ-R total score (63.87 ± 19.12 vs. 75.94 ± 12.25, *p* = 0.017) among normal-weight and overweight FMS were observed. For the healthy controls, we only observed that sleep quality was significantly poorer in obese women than in overweight women (*p* < 0.001).

### 3.3. Body Composition and VAS, TPC, FIQ-R, Fatigue, Sleep, and Anxiety

Beta estimates and 95% CI for body composition parameters and VAS, TPC, FIQ-R, fatigue, sleep quality, and anxiety in the FMS women and the healthy controls are presented in Table 3 and Table 4, respectively. Linear analysis regression revealed significant associations among FIQ-R.2 [β(95% CI) = 0.336, (0.027, 0.645), *p* = 0.034], FIQ-R.3 [β(95% CI) = 0.235, (0.017, 0.453), *p* = 0.035], and FIQ-R total score [β(95% CI) = 0.110, (0.010, 0.209), *p* = 0.032], and BMI after adjusting for age and menopause status in FMS women. Furthermore, an association between sleep latency and fat mass percentage adjusted for covariates was identified in the FMS patients [β (95% CI) = 1.910, (0.078, 3.742), *p* = 0.041]. Regarding the healthy controls, there were significant associations among fat mass and TPC [β(95% CI) = 0.884, (0.066, 1.703), *p* = 0.035] and reduced motivation assessed by MFI [β(95% CI) = −0.813, (−1.610, −0.016), *p* = 0.046] after adjusting by age and menopause status. Moreover, an association between sleep quality and visceral fat was identified β (95% CI) = 2.614, (2.192, 3.036), *p* = 0.008].

## 4. Discussion

In this study we investigated the associations among several symptoms related to FMS and BMI and body composition by assessing fat mass, fat mass percentage, and visceral fat in a population of women with FMS and healthy controls. We found that BMI was significantly associated with disease severity assessed by FIQ-R. We also evidenced that overweight and obese women have poor FIQ-R scores than normal-weight women, supporting the negative effect of increased body weight on FMS. Furthermore, our results revealed that fat mass percentage is associated with sleep latency in FMS women, and visceral fat is linked to sleep quality in healthy controls, suggesting a deleterious effect of obesity on sleep characteristics in both FMS and healthy women. Additionally, we showed that fat mass was associated with TPC and reduced motivation as assessed by the MFI, supporting the negative role of increased fat mass also in healthy women.

Obesity is a major concern for coexisting with FMS [6]. In our study sample, the prevalence of overweight/obesity in FMS women was 69.9% and the mean BMI was 29.11 kg/m^2^. Similar results were reported by Neumman et al., Okifuji et al., Kim et al., Aparicio et al., and Cordero et al. [8,15,16,18,21]. In a survey conducted in 2569 FMS patients, 70% of patients had a BMI of 25 kg/m^2^ and 43% had a BMI of 30 kg/m^2^ [47].

The role of BMI in the disease severity has been increasingly recognized [16,19,21,23]. Our results are consistent with prior research indicating that obese FMS patients have poor FIQ total scores. Aparicio et al. showed a positive relationship between BMI and FIQ subscales [18], and Kim et al. reported that groups with a higher BMI had more fibromyalgia-related symptoms and poor FIQ total scores, as well as poor scores in the FIQ subscales [16]. A recent study showed that BMI was positively correlated with FIQ in females with FMS [19]. A randomized controlled trial (RCT) also reported that weight loss in obese patients with FMS leads to significant improvements in the quality of life as shown by the decrease in the FIQ score [24]. Interestingly, the significant relationship between BMI and fibromyalgia impact has been proposed to be fully accounted for by physical factors and not by psychological factors, supporting the importance of counseling patients on physical factors (i.e., physical activity) that could improve the patients’ symptom experience [23]. Therefore, on the basis of our data and previous evidence, it should be stated that the management of FMS may include strategies to promote an optimal body weight among FMS patients.

Sleep disturbance has been postulated as a potential link between obesity and FMS [26]. In fact, there is evidence that indicates an association between sleep quality and FMS, supporting that the presence of obesity may be involved with sleepiness in these patients [8,21,24,26]. Additionally, sleep problems have been reported to play a critical role in exacerbating FMS symptoms since sleep may predict subsequent pain in FMS patients [48]. We revealed that FMS patients with higher fat mass percentage have longer sleep latency, and therefore poorer sleep quality. Likewise, in healthy women, visceral fat was associated with sleep quality. These results support the negative effect of obesity on sleep characteristics in both FMS and healthy women. Although we reported for the first time this association in FMS patients, in the general population evidence supports the positive relationship between fat mass and sleep latency [49]. Since this association has not been published in a population of FMS patients, these findings should be considered as preliminary and need to be validated in independent studies. Nevertheless, in line with our study, Cordero et al. found an association between BMI and morning tiredness, and Okifuji et al. reported that obesity was related to shorter sleep duration and increased restlessness during sleep in FMS patients [8,21]. Similarly, Senna et al. indicated that patients with FMS who lost weight had better sleep quality [24], and de Araújo et al. observed that the presence of obesity might be involved with sleepiness in FMS patients [26]. However, it should be noted that none of the previous work has tested the association with fat mass, and therefore further prospective studies will be required to establish the cause and effect relationship between fat mass and sleep quality in FMS.

We also reported a lack of relationships among pain score, TPC, fatigue, and anxiety and the BMI and body composition parameters. This finding is consistent with that reported by Yunus et al., where associations between BMI and the number of tender points and anxiety, as well as fatigue, were not statistically significant in a cohort of female patients with FMS [20]. Similarly, Zahorska-Markiewicz et al. did not observe any significant relationship between BMI and TPC in obese groups as compared with a normal weight group [50]. However, it should be noted that our study found that fat mass was associated with TPC in healthy women. In addition, Okifuji et al. supported that obesity may be a risk factor for having pain in the general population [9]. Interestingly, previous evidence has reported that the relationship between obesity and pain is not direct and might be modulated by several factors including inflammatory mediators, structural changes associated with obesity, and lifestyle characteristics [9]. Therefore, the lack of association between TPC and obesity parameters in FMS women may be explained by the fact that the nature of this relationship is not direct, and therefore several interacting factors might exert a major contribution. It could be hypothesized that in patients with low-pressure pain thresholds and a high number of tender points, such as FMS patients, factors other than obesity could also play a relevant role. On the other hand, the lack of a relationship between obesity and fatigue observed in our study may be due to different reasons. First, previous evidence has indicated that sleep quality is related to metabolic factors such as obesity, whereas, fatigue appears to be related to psychological distress [51]. This fact could explain why obesity is associated with sleep quality and not with fatigue. Secondly, it should be noted that fatigue was estimated by a self-assessment in our study. However, there are different types of fatigue measures including objective physical (posturography), objective mental (psychomotor vigilance task), subjective physical and mental (self-assessment), and objective and subjective realistic (oculomotor behavior, observer-rated facial expression, typing performance), and therefore subjective assessment of fatigue based on patient-reported outcomes has inherent limitations [52]. Further studies are needed to elucidate the relationship between obesity and objective measures of fatigue in FMS patients.

This study has some limitations. First, due to its cross-sectional nature, casual relationships have not been established. In addition, since sleep quality was assessed by the PSQI, which is a self-perceived questionnaire, recall bias is a inherent concern. An objective measure, such as polysomnography, is considered the gold standard to assess sleep quality [53]. However, it may not be feasible to carry this out in epidemiological studies with large study cohorts. Furthermore, PSQI is one of the most widely used and recommended instruments to measure sleep quality [41]. Secondly, our study sample consisted of a well-characterized population of FMS women, and therefore our data might not be generalizable to other populations. Another potential limitation of this study is that a possible selection bias may exist, due to the control group being recruited on a convenience sample. In addition, given the higher number of statistical interactions that have been tested, we have not eliminated the possibility that multiple testing could play a role in our results. However, the direction of the significant findings for the FIQ-R outcomes make it unlikely that our results could be explained by chance alone. Finally, the associations between FMS-related clinical symptoms assessed by the FIQ-R and obesity measurements were shown only in FMS cases, since the FIQ-R is a specific instrument for the evaluation of FMS patients and could not be evaluated in control. Therefore, we cannot discard that the association between BMI and FMS symptoms might be specific to FMS patients. Despite its limitations, the present study still has a number of strengths. To the best of our knowledge, this is the first study to examine the associations between body composition measurements including fat mass percentage and visceral fat and FMS-related symptoms in FMS patients.

## 5. Conclusions

In conclusion, our findings revealed that higher BMI values are associated with poor FIQ-R scores and that overweight and obese women with FMS have higher symptom severity. However, further work is required to validate whether this relationship is specific for FMS patients. The promotion of an optimal BMI might contribute to ameliorate some of the FMS symptoms. Thus, development and implementation of obesity prevention programs based on a balanced diet and increased physical activity to improve the severity of symptoms in FMS women are of special interest.

## Figures and Tables

**Table 1:** Physical characteristics of women with fibromyalgia and healthy controls.

	Cases (*n* = 73)	Controls (*n* = 73)	*p* Value
Age (years)	56.52 ± 7.49	57.40 ± 10.74	0.568
Height (cm)	158.56 ± 5.89	158.26 ± 6.44	0.769
Weight (kg)	71.87 ± 12.38	70.52 ± 11.52	0.497
BMI (kg/m^2^)	29.11 ± 6.01	28.22 ± 4.61	0.317
Body composition			
% Fat mass	37.48 ± 6.46	37.28 ± 5.86	0.842
Fat mass (gr)	27.66 ± 9.54	26.82 ± 8.45	0.581
Visceral fat	8.99 ± 3.06	8.93 ± 2.95	0.913
TPC	17.56 ± 1.26	1.87 ± 3.46	<0.001
VAS	7.40 ± 1.75	1.77 ± 2.62	<0.001
FIQ-R			
FIQ-R.1	20.04 ± 5.39	-	
FIQ-R.2	13.92 ± 4.83	-	
FIQ-R.3	39.06 ± 6.96	-	
Total score	73.08 ± 14.73	-	
Fatigue			
MFI general fatigue	18.25 ± 2.22	9.76 ± 4.24	<0.001
MFI physical fatigue	16.52 ± 2.75	9.38 ± 4.45	<0.001
MFI mental fatigue	15.15 ± 1.65	11.58 ± 2.92	<0.001
MFI reduced activity	15.05 ± 3.86	7.94 ± 4.28	<0.001
MFI reduced motivation	14.36 ± 3.49	7.68 ± 3.49	<0.001
Fatigue total score	79.33 ± 9.44	46.35 ± 15.64	<0.001
Sleep Quality Index			
Sleep quality	2.31 ± 0.77	1.29 ± 0.75	0.010
Sleep latency	2.30 ± 0.91	0.57 ± 0.78	0.001
Sleep duration	2.12 ± 0.90	1.00 ± 0.57	0.001
Habitual sleep efficiency	2.05 ± 1.07	0.14 ± 0.37	<0.001
Sleep disturbances	2.29 ± 0.48	1.29 ± 0.48	0.001
Sleeping medication	2.01 ± 1.33	0.57 ± 1.13	0.014
Daytime dysfunction	2.30 ± 0.80	0.43 ± 0.53	<0.001
Global score	15.44 ± 3.81	5.29 ± 2.43	<0.001
Anxiety	33.11 ± 9.59	11.56 ± 10.19	<0.001

BMI: body mass index, TPC: tender points count, VAS: visual analog scale, FIQ-R: revised Fibromyalgia Impact Questionnaire, FIQ-R.1: level of activity, FIQ-R.2: global impact, FIQ-R.3: symptoms intensity, MFI: multidimensional fatigue inventory. Numerical variables are shown as mean ± SD (Standard Deviation).

**Table 2:** Comparison of clinical data between normal weight and overweight/obese women with fibromyalgia and healthy women.

	Cases (*n* = 73)			Controls (*n* = 73)		
	Normal (*n* = 17)	Overweight/Obese (*n* = 56)	*p* Value	Normal (*n* = 21)	Overweight/Obese (*n* = 52)	*p* Value
VAS	6.93 ± 2.28	7.57 ± 1.59	0.499	1.93 ± 2.52	1.68 ± 2.73	0.650
TPC	17.80 ± 0.77	17.46 ± 1.41	0.590	1.33 ± 3.17	1.90 ± 3.72	0.257
FIQ-R						
FIQ-R.1	16.82 ± 6.86	20.66 ± 4.71	0.030	-	-	
FIQ-R.2	11.60 ± 4.96	14.89 ± 4.43	0.059	-	-	
FIQ-R.3	35.20 ± 89.02	40.33. ± 5.60	0.033	-	-	
Total score	63.87 ± 19.12	75.94 ± 12.25	0.017	-	-	
Fatigue						
MFI general fatigue	18.73 ± 1.28	18.15 ± 2.40	0.421	9.27 ± 4.51	9.50 ± 4.03	0.918
MFI physical fatigue	16.13 ± 3.06	16.61 ± 2.72	0.614	8.73 ± 4.75	9.60 ± 3.90	0.690
MFI mental fatigue	15.47 ± 1.45	15.24 ± 1.55	0.937	10.93 ± 2.12	11.57 ± 3.42	0.311
MFI reduced activity	13.47 ± 4.47	15.43 ± 3.64	0.150	7.87 ± 4.27	7.73 ± 4.29	0.984
MFI reduced motivation	14.00 ± 3.56	14.59 ± 3.48	0.566	8.40 ± 4.77	7.20 ± 2.68	0.253
Fatigue total score	77.80 ± 9.44	80.02 ± 9.61	0.449	45.20 ± 18.60	45.60 ± 14.22	0.939
Sleep Quality Index						
Sleep quality	2.40 ± 0.73	2.35 ± 0.71	0.888	0.67 ± 0.57	2.00 ± 0.83	<0.001
Sleep latency	2.07 ± 1.10	2.46 ± 0.73	0.120	0.33 ± 0.57	2.00 ± 2.41	0.485
Sleep duration	2.27 ± 0.96	2.19 ± 0.79	0.785	1.00 ± 0.00	0.50 ± 0.70	0.357
Habitual sleep efficiency	2.07 ± 1.10	2.13 ± 1.05	0.972	0.33 ± 0.57	0.00 ± 0.00	0.969
Sleep disturbances	2.40 ± 0.50	2.26 ± 0.48	0.182	1.00 ± 0.00	1.50 ± 0.70	0.357
Sleeping medication	2.00 ± 1.36	2.08 ± 1.32	0.853	1.00 ± 1.73	0.50 ± 0.70	0.875
Daytime dysfunction	2.20 ± 0.67	2.35 ± 0.82	0.289	0.67 ± 0.57	0.50 ± 0.70	0.181
Global score	15.40 ± 4.30	15.95 ± 3.22	0.712	5.00 ± 3.60	6.00 ± 2.82	0.539
Anxiety	31.33 ± 1126	33.78 ± 9.35	0.441	11.33 ± 9.74	11.23 ± 11.07	0.893

VAS: visual analog scale, TPC: tender points count, FIQ-R: revised Fibromyalgia Impact Questionnaire, MFI: multidimensional fatigue inventory, FIQ-R.1: level of activity, FIQ-R.2: global impact, FIQ-R.3: symptoms intensity. Numerical variables are shown as mean ± SD.

**Table 3:** Associations among body composition, VAS, TPC, FIQ-R, fatigue, sleep, and anxiety in women with FMS.

	BMI		% Fat Mass		Fat Mass		Visceral Fat	
	β (95% CI)	*p* Value	β (95% CI)	*p* Value	β (95% CI)	*p* Value	β (95% CI)	*p* Value
VAS	0.226 (−0.619, 1.070)	0.595	−0.074 (−0.947, 0.799)	0.866	0.106 (−1.203, 1.415)	0.872	0.011 (−0.359, 0.381)	0.952
TPC	0.081 (−1.049, 1.211)	0.887	−0.217 (−1.382, 0.948)	0.711	−0.208 (−1.955, 1.540)	0.813	−0.007 (−0.502, 0.487)	0.977
FIQ-R								
FIQ-R.1	0.194 (−0.072, 0.459)	0.150	0.118 (−0159, 0.395)	0.398	0.168 (−0.247, 0.584)	0.421	0.050 (−0.067, 0.167)	0.399
FIQ-R.2	0.336 (0.027, 0.645)	0.034	0.247 (−0.077, 0572)	0.133	0.350 (−0137, 0.838)	0.156	0.100 (−0.038, 0.238)	0.153
FIQ-R.3	0.235 (0.017, 0.453)	0.035	0.130 (−0.100, 0.361)	0.262	0.275 (−0.067, 0.617)	0.113	0.077 (−0.020, 0.173)	0.120
Total Score	0.110 (0.010, 0.209)	0.032	0.069 (−0.037, 0.174)	0.198	0.117 (−0.040, 0.274)	0.143	0.033 (−0.011, 0.078)	0.142
Fatigue								
MFI general fatigue	0.243 (−0.412, 0.898)	0.461	0.083 (−0.595, 0.761)	0.808	0.293 (−0.722, 1.307)	0.567	0.088 (−0.199, 0.375)	0.543
MFI physical fatigue	0.442 (−0.068, 0.953)	0.088	0.163 (−0.374, 0.700)	0.547	0.264 (−0.541, 1.069)	0.514	0.093 (−0.134, 0.320)	0.415
MFI mental fatigue	−0.852 (−1.822, 0.118)	0.084	−0.662 (−1673, 0.350)	0.196	−1.348 (−2.848, 0.151)	0.077	−0.414 (−0.836, −0.008)	0.055
MFI reduced activity	0.236 (−0.137, 0.609)	0.211	0.314 (−0.068, 0.695	0.105	0.466 (−0.106, 1.039)	0.108	0.118 (−0.044, 0.281)	0.150
MFI reduced motivation	0.164 (−0.267, 0.595)	0.449	0.070 (−0.376, 0.516)	0.756	0.080 (−0.589, 0.749)	0.812	0.055 (−0.134, 0.244)	0.561
Fatigue total score	0.090 (−0.061, 0.242)	0.238	0.063 (−0.094, 0.220)	0.425	0.093 (−0.142, 0.329)	0.432	0.029 (−1.037, 0.096)	0.382
Sleep Quality Index								
Sleep quality	−0.449 (−2.589, 1.691)	0.677	0.372 (−1.825, 2.569)	0.736	0.795 (−2.504, 4.094)	0.632	0.148 (−0.789, 1.084)	0.753
Sleep latency	0.474 (−1.368, 2.315)	0.609	1.910 (0.078, 3.742)	0.041	2.744 (−0.019, 5.506)	0.052	0.586 (−0.206, 1.378)	0.144
Sleep duration	−1295 (−3.167, 0.577)	0.171	−1.401 (−3.328, 0.526)	0.151	−1.753 (−4.685, 1.178)	0.236	−0.608 (−1.424, 0208)	0.141
Habitual sleep efficiency	−0.561 (−2.130, 1.008)	0.477	−0.738 (−2.355, 0.880)	0.365	−0.838 (−3.295, 1.620)	0.498	−0.339 (−1.022, 0.345)	0.325
Sleep disturbances	2.434 (−0.651, 5.520)	0.120	−0.576 (−3.806, 2.655)	0.723	1.316 (−3.534, 6.166)	0.589	0.227 (−1.147, 1.600)	0.743
Sleeping medication	0.792 (−0.403, 1.986)	0.190	0.301 (−0.941, 1.542)	0.630	0.636 (−1.225, 2.498)	0.497	0.170 (−0.358, 0.697)	0.523
Daytime dysfunction	1.165 (−0.779, 3.109)	0.236	1.448 (−0.536, 3.432)	0.150	2.682 (−0.273, 5.636)	0.074	0.621 (−0.224, 1.467)	0.147
Global score	0.179 (−0.299, 0.657)	0.456	0.143 (−0.352, 0.638)	0.565	0.370 (−0.376, 1.115)	0.325	0.071 (−0.138, 0.280)	0.500
Anxiety	0.107 (−0.045, 0.259)	0.163	0.015 (−1.444, 0.174)	0.855	0.095 (−0.142, 0.333)	0.425	0.031 (−0.036, 0.098)	0.363

BMI: body mass index, VAS: visual analog scale, TPC: tender points count, FIQ-R: revised Fibromyalgia Impact Questionnaire, MFI: multidimensional fatigue inventory, FIQ-R.1: level of activity, FIQ-R.2: global impact, FIQ-R.3: symptoms intensity. Linear regression models were adjusted for the covariates age and menopausal state.

**Table 4:** Associations among body composition, VAS, TPC, FIQ-R, fatigue, sleep, and anxiety in healthy women.

	BMI		% Fat Mass		Fat Mass		Visceral Fat	
	β (95% CI)	*p* Value	β (95% CI)	*p* Value	β (95% CI)	*p* Value	β (95% CI)	*p* Value
VAS	−0.150 (−0.738, 0.420)	0.583	−0.335 (−1.028, 0.359)	0.335	−0.505 (−1.566, 0.558)	0.344	−0.125 (−0.429, 0.179)	0.412
TPC	0.324 (−0.135, 0.782)	0.161	0.380 (−0.174, 0.933)	0.173	0.884 (0.066, 1.703)	0.035	0.191 (−0.049, 0.432)	0.116
Fatigue								
MFI general fatigue	−0.0.76 (−0.453, 0.301)	0.687	−0.273 (−0.720, 0.174)	0.225	−0.398 (−1.082, 0.287)	0.247	−0.109 (−0.306, 0.087)	0.267
MFI physical fatigue	0.089 (−0.289, 0.467)	0.638	−0.228 (−0.679, 0.224)	0.314	−0.161 (−0.858, 0.535)	0.642	−0.040 (−0.240, 0.160)	0.687
MFI mental fatigue	−0.119 (0.654, 0.416)	0.655	−0.283 (−0.923, 0.358)	0.378	−0.534 (−1.508, 0.439)	0.274	−0.151 (−0.430, 0.128)	0.280
MFI reduced activity	0.015 (−0.360, 0.390)	0.937	−0.312 (−0.753, 0.129)	0.161	−0.308 (−0.992, 0.375)	0.367	−0.080 (−0.276, 0.116)	0.416
MFI reduced motivation	−0.313 (-0.757, 0.131)	0.162	−0.488 (−1.015, 0.038)	0.068	−0.813 (−1.610, −0.016)	0.046	−0.224 (−0.453, 0.006)	0.056
Fatigue total score	−0.018 (−0.118, 0.083)	0.726	−0.091 (−0.209, 0.026)	0.124	−0.120 (−0.301, 0.061)	0.188	−0.032 (−0.084, 0.020)	0.214
Sleep Quality Index								
Sleep quality	3.674 (−0.503, 7.851)	0.057	8.190 (−1.303, 17.684)	0.058	9.329 (−7.253, 25.911)	0.088	2.614 (2.192, 3.036)	0.008
Sleep latency	2.660 (−35.889, 41.209)	0.542	5.919 (−80.166, 92.005)	0.543	6.400 (−96.221, 109.020)	0.573	2.094 (−22.646, 26.834)	0.477
Sleep duration	−17.53 (−131.095, 96.019)	0.300	−39.140 (−291.489, 213209)	0.299	−45.870 (−307.231, 215.491)	0.268	−11.720 (−107.85, 84.413)	0.365
Habitual sleep efficiency	−1.174 (−108.57, 106.230)	0.912	−2.650 (−242.079, 236.778)	0.911	−4.068 (−276136, 268.000)	0.880	−0.218 (−77.016, 76.580)	0.977
Sleep disturbances	17.538 (−96.019, 131.095)	0.300	39.140 (−213.209, 291.489)	0.299	45.870 (−215.491, 307.231)	0.268	11.720 (−84.413, 107.853)	0.365
Sleeping medication	0.278 (−32.486, 33.042)	0.932	0.611 (−72.459, 73.681)	0.933	0.383 (−83.640, 84.405)	0.963	0.382 (−22.462, 23.226)	0.867
Daytime dysfunction	9.660 (−38.406, 57.726)	0.238	21.525 (−86.124, 129.174)	0.239	24.160 (−114.031, 162.351)	0.269	7.083 (−17.951, 32.118)	0.173
Global score	0.641 (−10.420, 11.701)	0.596	1.425 (−23268, 26.119)	0.597	1.524 (−27.722, 30.770)	0.628	0.515 (−6.696, 7.725)	0.531
Anxiety	0.018 (−0.133, 0.170)	0.809	−0.035 (−0.216, 0.147)	0.701	−0.086 (−0.364, 0.193)	0.538	−0.016 (−0.095, 0.064)	0.693

BMI: body mass index, VAS: visual analog scale, TPC: tender points count, FIQ-R: revised Fibromyalgia Impact Questionnaire, MFI: multidimensional fatigue inventory. Linear regression models were adjusted for the covariates age and menopausal state.

## References

[1] Bellato E., Marini E., Castoldi F., Barbasetti N., Mattei L., Bonasia D.E., Blonna D. (2012). Fibromyalgia syndrome: Etiology, pathogenesis, diagnosis, and treatment. Pain Res. Treat..

[2] La Rubia M., Rus A., Molina F., Del Moral M.L. (2013). Is fibromyalgia-related oxidative stress implicated in the decline of physical and mental health status?. Clin. Exp. Rheumatol..

[3] Sluka K.A., Clauw D.J. (2016). Neurobiology of fibromyalgia and chronic widespread pain. Neuroscience.

[4] Tanriverdi F., Karaca Z., Unluhizarci K., Kelestimur F. (2007). The hypothalamo–pituitary–adrenal axis in chronic fatigue syndrome and fibromyalgia syndrome. Stress.

[5] Staud R. (2015). Cytokine and immune system abnormalities in fibromyalgia and other central sensitivity syndromes. Curr. Rheumatol. Rev..

[6] Ursini F., Naty S., Grembiale R.D. (2011). Fibromyalgia and obesity: The hidden link. Rheumatol. Int..

[7] Hruby A., Hu F.B. (2015). The Epidemiology of Obesity: A Big Picture. Pharmacoeconomics.

[8] Okifuji A., Donaldson G.W., Barck L., Fine P.G. (2010). Relationship between fibromyalgia and obesity in pain, function, mood, and sleep. J. Pain Off. J. Am. Pain Soc..

[9] Okifuji A., Hare B.D. (2015). The association between chronic pain and obesity. J. Pain Res..

[10] Stone A.A., Broderick J.E. (2012). Obesity and Pain Are Associated in the United States. Obesity.

[11] Bose M., Oliván B., Laferrère B. (2009). Stress and obesity: The role of the hypothalamic-pituitary-adrenal axis in metabolic disease. Curr. Opin. Endocrinol. Diabetes Obes..

[12] Asghar A., Sheikh N. (2017). Role of immune cells in obesity induced low grade inflammation and insulin resistance. Cell. Immunol..

[13] Schmidt F.M., Weschenfelder J., Sander C., Minkwitz J., Thormann J., Chittka T., Mergl R., Kirkby K.C., Faßhauer M., Stumvoll M. (2015). Inflammatory Cytokines in General and Central Obesity and Modulating Effects of Physical Activity. PLoS ONE.

[14] Rus A., Molina F., Gassó M., Camacho M.V., Peinado M.Á., del Moral M.L. (2016). Nitric Oxide, Inflammation, Lipid Profile, and Cortisol in Normal- and Overweight Women with Fibromyalgia. Biol. Res. Nurs..

[15] Neumann L., Lerner E., Glazer Y., Bolotin A., Shefer A., Buskila D. (2008). A cross-sectional study of the relationship between body mass index and clinical characteristics, tenderness measures, quality of life, and physical functioning in fibromyalgia patients. Clin. Rheumatol..

[16] Kim C.H., Luedtke C.A., Vincent A., Thompson J.M., Oh T.H. (2012). Association of body mass index with symptom severity and quality of life in patients with fibromyalgia. Arthritis Care Res..

[17] Gota C.E., Kaouk S., Wilke W.S. (2015). Fibromyalgia and Obesity. J. Clin. Rheumatol..

[18] Aparicio V.A., Ortega F.B., Carbonell-Baeza A., Gatto-Cardia C., Sjöström M., Ruiz J.R., Delgado-Fernández M. (2013). Fibromyalgia’s Key Symptoms in Normal-Weight, Overweight, and Obese Female Patients. Pain Manag. Nurs..

[19] Koçyiğit B.F., Okyay R.A. (2018). The relationship between body mass index and pain, disease activity, depression and anxiety in women with fibromyalgia. PeerJ.

[20] Yunus M.B., Arslan S., Aldag J.C. (2002). Relationship between body mass index and fibromyalgia features. Scand. J. Rheumatol..

[21] Cordero M.D., Alcocer-Gómez E., Cano-García F.J., Sánchez-Domínguez B., Fernández-Riejo P., Moreno Fernández A.M., Fernández-Rodríguez A., De Miguel M. (2014). Clinical symptoms in fibromyalgia are associated to overweight and lipid profile. Rheumatol. Int..

[22] Timmerman G.M., Calfa N.A., Stuifbergen A.K. (2013). Correlates of Body Mass Index in Women With Fibromyalgia. Orthop. Nurs..

[23] Vincent A., Clauw D., Oh T.H., Whipple M.O., Toussaint L.L. (2014). Decreased physical activity attributable to higher body mass index influences fibromyalgia symptoms. PM R.

[24] Senna M.K., Sallam R.A.E.R., Ashour H.S., Elarman M. (2012). Effect of weight reduction on the quality of life in obese patients with fibromyalgia syndrome: A randomized controlled trial. Clin. Rheumatol..

[25] Segura-Jiménez V., Castro-Piñero J., Soriano-Maldonado A., Álvarez-Gallardo I.C., Estévez-López F., Delgado-Fernández M., Carbonell-Baeza A. (2016). The association of total and central body fat with pain, fatigue and the impact of fibromyalgia in women; role of physical fitness. Eur. J. Pain.

[26] De Araújo T.A., Mota M.C., Crispim C.A. (2015). Obesity and sleepiness in women with fibromyalgia. Rheumatol. Int..

[27] Diaz-Piedra C., Di Stasi L.L., Baldwin C.M., Buela-Casal G., Catena A. (2015). Sleep disturbances of adult women suffering from fibromyalgia: Asystematic review of observational studies. Sleep Med. Rev..

[28] Choy E.H.S. (2015). The role of sleep in pain and fibromyalgia. Nat. Rev. Rheumatol..

[29] Stuifbergen A.K., Phillips L., Carter P., Morrison J., Todd A. (2010). Subjective and objective sleep difficulties in women with fibromyalgia syndrome. J. Am. Acad. Nurse Pract..

[30] Landis C.A., Frey C.A., Lentz M.J., Rothermel J., Buchwald D., Shaver J.L.F. (2003). Self-reported sleep quality and fatigue correlates with actigraphy in midlife women with fibromyalgia. Nurs. Res..

[31] Sabeti E., Gryak J., Derksen H., Biwer C., Ansari S., Isenstein H., Kratz A., Najarian K., Sabeti E., Gryak J. (2019). Learning Using Concave and Convex Kernels: Applications in Predicting Quality of Sleep and Level of Fatigue in Fibromyalgia. Entropy.

[32] Arout C.A., Sofuoglu M., Bastian L.A., Rosenheck R.A. (2018). Gender Differences in the Prevalence of Fibromyalgia and in Concomitant Medical and Psychiatric Disorders: A National Veterans Health Administration Study. J. Women’s Health.

[33] Wolfe F., Hassett A.L., Walitt B., Michaud K. (2011). Mortality in fibromyalgia: A study of 8186 patients over thirty-five years. Arthritis Care Res..

[34] Wolfe F., Cathey M.A. (1985). The epidemiology of tender points: A prospective study of 1520 patients. J. Rheumatol..

[35] Buskila D., Neumann L., Alhoashle A., Abu-Shakra M. (2000). Fibromyalgia syndrome in men. Semin. Arthritis Rheum..

[36] Chesterton L.S., Sim J., Wright C.C., Foster N.E. (2007). Interrater Reliability of Algometry in Measuring Pressure Pain Thresholds in Healthy Humans, Using Multiple Raters. Clin. J. Pain.

[37] Vanderweeën L., Oostendorp R.A.B., Vaes P., Duquet W. (1996). Pressure algometry in manual therapy. Man. Ther..

[38] Jones D.H., Kilgour R.D., Comtois A.S. (2007). Test-Retest Reliability of Pressure Pain Threshold Measurements of the Upper Limb and Torso in Young Healthy Women. J. Pain.

[39] Marques A.P., Assumpção A., Matsutani L.A., Pereira C.A.B., Lage L. (2008). Pain in fibromyalgia and discrimination power of the instruments: Visual Analog Scale, Dolorimetry and the McGill Pain Questionnaire. Acta Reumatol. Port..

[40] Salgueiro M., García-Leiva J.M., Ballesteros J., Hidalgo J., Molina R., Calandre E.P. (2013). Validation of a Spanish version of the Revised Fibromyalgia Impact Questionnaire (FIQR). Health Qual. Life Outcomes.

[41] Hita-Contreras F., Martínez-López E., Latorre-Román P.A., Garrido F., Santos M.A., Martínez-Amat A. (2014). Reliability and validity of the Spanish version of the Pittsburgh Sleep Quality Index (PSQI) in patients with fibromyalgia. Rheumatol. Int..

[42] Munguía-Izquierdo D., Segura-Jiménez V., Camiletti-Moirón D., Pulido-Martos M., Alvarez-Gallardo I.C., Romero A., Aparicio V.A., Carbonell-Baeza A., Delgado-Fernández M. (2012). Multidimensional Fatigue Inventory: Spanish adaptation and psychometric properties for fibromyalgia patients. The Al-Andalus study. Clin. Exp. Rheumatol..

[43] Beyazal M.S., Tüfekçi A., Kırbaş S., Topaloğlu M.S. (2018). The Impact of Fibromyalgia on Disability, Anxiety, Depression, Sleep Disturbance, and Quality of Life in Patients with Migraine. Arch. Neuropsychiatry.

[44] Vázquez Morejón A.J., Vázquez-Morejón Jiménez R., Zanin G.B. (2014). Beck Anxiety Inventory: Psychometric characteristics in a sample from the clinical Spanish population. Span. J. Psychol..

[45] Ghasemi A., Zahediasl S. (2012). Normality tests for statistical analysis: A guide for non-statisticians. Int. J. Endocrinol. Metab..

[46] Kim H.-Y. (2018). Statistical notes for clinical researchers: Analysis of covariance (ANCOVA). Restor. Dent. Endod..

[47] Bennett R.M., Jones J., Turk D.C., Russell I.J., Matallana L. (2007). An internet survey of 2596 people with fibromyalgia. BMC Musculoskelet. Disord..

[48] Bigatti S.M., Hernandez A.M., Cronan T.A., Rand K.L. (2008). Sleep disturbances in fibromyalgia syndrome: Relationship to pain and depression. Arthritis Rheum..

[49] Wirth M.D., Hébert J.R., Hand G.A., Youngstedt S.D., Hurley T.G., Shook R.P., Paluch A.E., Sui X., James S.L., Blair S.N. (2015). Association between actigraphic sleep metrics and body composition. Ann. Epidemiol..

[50] Zahorska-Markiewicz B., Zych P., Kucio C. (1988). Pain sensitivity in obesity. Acta Physiol. Pol..

[51] Vgontzas A.N., Bixler E.O., Chrousos G.P. (2006). Obesity-Related Sleepiness and Fatigue: The Role of the Stress System and Cytokines. Ann. N. Y. Acad. Sci..

[52] Völker I., Kirchner C., Bock O.L. (2016). On the relationship between subjective and objective measures of fatigue. Ergonomics.

[53] Ibáñez V., Silva J., Cauli O. (2018). A survey on sleep assessment methods. PeerJ.

